# Stimulation of lymphocyte anti-melanoma activity by co-cultured macrophages activated by complex homeopathic medication

**DOI:** 10.1186/1471-2407-9-293

**Published:** 2009-08-22

**Authors:** Fernando SF Guimarães, Ana PR Abud, Simone M Oliveira, Carolina C Oliveira, Beatriz César, Lucas F Andrade, Lucélia Donatti, Juarez Gabardo, Edvaldo S Trindade, Dorly F Buchi

**Affiliations:** 1 Departamento de Biologia Celular, Laboratório de Pesquisa em Células Inflamatórias e Neoplásicas, Universidade Federal do Paraná (UFPR), Curitiba – PR, Brazil

## Abstract

**Background:**

Melanoma is the most aggressive form of skin cancer, and the most rapidly expanding cancer in terms of worldwide incidence. Chemotherapeutic approaches to treat melanoma have been uniformly disappointing. A Brazilian complex homeopathic medication (CHM), used as an immune modulator, has been recommended for patients with depressed immune systems. Previous studies in mice have demonstrated that the CHM activates macrophages, induces an increase in the number of leukocytes and improves the murine response against Sarcoma-180.

**Methods:**

Here we studied the interaction of mouse lymph node lymphocytes, co-cultured *in vitro *with macrophages in the presence or absence of the CHM, with B16F10 melanoma cells.

**Results:**

Lymphocytes co-cultured with macrophages in the presence of the CHM had greater anti-melanoma activity, reducing melanoma cell density and increasing the number of lysed tumor cells. There was also a higher proportion of activated (CD25^+^) lymphocytes with increased viability. Overall, lymphocytes activated by treatment destroyed growing cancer cells more effectively than control lymphocytes.

**Conclusion:**

Co-culture of macrophages with lymphocytes in the presence of the CHM enhanced the anti-cancer performance of lymphocytes against a very aggressive lineage of melanoma cells. These results suggest that non-toxic therapies using CHMs are a promising alternative approach to the treatment of melanomas. In addition, they are attractive combination-therapy candidates, which may enhance the efficacy of conventional medicines by improving the immune response against tumor cells.

## Background

Melanoma represents a significant worldwide public health risk, and from the standpoint of incidence, constitutes the fastest growing of all cancer types. Malign melanoma is the most aggressive form of skin cancer, with a mortality rate that has risen about 2% annually since 1960. Although early stage melanoma can be cured surgically, once melanoma metastasizes to major organs (stage IV), it is virtually incurable. Patients with advanced melanoma have a median survival time of less than one year, and the estimated 5-year survival rate is less than 15% [1].

Melanoma has aggressive metastatic potential, particularly to lymph nodes, lung and brain. Reports of metastases to other organs, including bone, pancreas, adrenals and small intestine, have also been described. There are few chemotherapeutic agents available for treating metastatic melanoma, and those that have been used have achieved uniformly disappointing results. No single chemotherapeutic agent currently offers a response rate greater than 25%, and treatment is invariably accompanied by significant side effects, including myelosuppression, nausea and emesis [1,2].

Immunotherapy remains the subject of intense investigation in both adjuvant and advanced disease settings, and attempts are being made to target melanoma defense mechanisms that blunt the effectiveness of host immune responses. However, there are few laboratory reports on homeopathic medicines in the medical literature; of those that have been published, most are clinical reports that have employed varied methodologies and yielded controversial, and often questionable, results. Despite this paucity of basic research, a large number of people continue to use such therapies. In Europe, homeopathy is the most frequent complementary and alternative medical therapy [3]. Here we describe the results of an experimental laboratory validation of the potential of peritoneal macrophages, challenged with a complex homeopathic medication (CHM), to stimulate the immune effectiveness of mesenteric lymph node lymphocytes (Ly). This new form of immunomodulatory therapy is based on Hahnemann's ancient homeopathic techniques, which use diluted substances that are vigorously shaken (succussed) during preparation.

Previous studies have demonstrated that CHM treatment activates macrophages both *in vivo *and *in vitro*, inducing several molecular modifications in macrophages. CHM treatment has been shown to suppress previously elevated levels of tumor necrosis factor-α (TNF-α), and increase the activity of NADPH oxidase and expression of inducible nitric oxide synthase (iNOS), resulting in increases in reactive oxygen species (ROS) and nitric oxide (NO), respectively [4,5]. In addition, a CHM has been shown to stimulate the endosomal/lysosomal system and increase the phagocytic activity of macrophages upon interaction with *Saccharomyces cerevisiae *and *Trypanosoma cruzi *epimastigotes [6]. CHM modulatory effects have also been observed with experimental infection by *Leishmania amazonensis and Paracoccidioides brasiliensis *both *in vivo *and *in vitro*, where it has been shown to limit infection progression and dissemination [7,8]. CHM treatment *in vivo *decreased IL-2 and IL-4 production, and cultures treated with CHM exhibited differential expression of 147 genes [9]. Moreover, CHM is neither toxic nor mutagenic [10]. Similarly, an improvement in the immune response of treated mice has been demonstrated in studies with Sarcoma 180. In these studies, there was a significant infiltration of lymphoid cells, granulation tissue and fibrosis developed and surrounded the tumor, and sarcoma size was reduced. All treated mice survived, and 30% exhibited a total regression of the tumor. Treatment increased the total numbers of leukocytes, specifically increasing B cells, NK cells and CD4+ T cells [11]. These results suggest a direct or indirect action of the medication on hematopoiesis. A subsequent microscopic study of bone marrow cells showed that monocytic lineage (CD11b) and stromal cells (adherent cells) were activated by treatment [12,13].

Melanoma cells are highly malignant and have a profound capacity to avoid detection by immune cells. Further, the CHM evaluated here activates the immune system by stimulating macrophages. Thus, we sought to verify the communication between activated macrophages and lymphocytes by assessing cell-cell interactions and anti-melanoma activity of lymphocytes co-cultured with macrophages in the presence of the CHM.

## Methods

### Complex homeopathic medication – CHM

The CHM represents a new form of immunomodulatory therapy and follows Hahnemann's ancient homeopathic techniques. Mother tinctures were purchased from authorized agencies sanctioned by the Brazilian Health Ministry. These agencies assure the quality (endotoxin free) and physico-chemical composition of their products. Starting from the original mother tincture – an ethanolic extract, in the case of a plant – several dynamizations/succussion (shaking) and serial dilutions in distilled water are performed. The CHM used to challenge macrophages is a complex matrix obtained from *Calcarea carbonica *CH5 with associations, comprised of a 10%–20% concentration of each compound obtained after vigorous shaking and five decimal dynamizations. The final solution contains *Aconitum napellus, Arsenicum album, Asa foetida, Calcarea carbonica, Conium maculatum, Ipecacuanha, Phosphorus, Rhus tox, Silicea, Sulphur*, and *Thuya occidentalis*, all in decimal dilutions of Hahnemann (dH) in distilled water. The resulting aqueous solution is colorless and odorless, and has an alcohol concentration < 1%. The medication was sterilized by filtration through 0.22 μm MILLEX GV Durapore PVDF membranes (Millipore), maintained at room temperature and vigorously shaken (succussed) immediately before each treatment.

### Animals

Macrophages and lymphocytes were harvested from 2–3-month-old male Swiss mice from the Central Animal House of the Universidade Federal do Paraná (UFPR). Experiments were performed at the Laboratório de Pesquisa em Células Neoplásicas e Inflamatórias, UFPR, which has a management program for produced residues. All recommendations of the National Law (No. 6.638, November, 5, 1979) for scientific management of animals were observed, and the Institutional Animal Care Committee of UFPR approved all related practices. All experiments were performed at least three times in triplicate, and data analysis was performed in a double-blinded manner.

### Cell culture

#### Macrophages

Cells were harvested from the peritoneal cavity with cold phosphate buffer saline (PBS), pH 7.4, and the number of cells was determined using a Neubauer chamber. The cells were plated in culture flasks and incubated at 37°C in a humidified atmosphere containing 5% CO_2_. After incubating for 15 min, non-adherent cells were removed by washing. The remaining adherent cells, mainly macrophages, were cultured with Dulbecco's modified eagle's medium (DMEM, GIBCO) supplemented with 10% heat-inactivated fetal bovine serum (FBS) containing 1 U/ml penicillin, 1 μg/ml streptomycin, and 2.5 μg/ml amphotericin (GIBCO), at 37°C in a humidified atmosphere containing 5% CO_2_.

#### Lymphocytes

Lymph nodes were removed from the mesentery. The tissue was dissociated using sterile Medicons (BD) and the cell suspension was filtered with a 100-μm mesh filter (Falcon, BD) to remove tissue fragments. After washing by centrifugation, the final suspension was incubated in a culture flask with PBS at 37°C in a humidified atmosphere containing 5% CO_2_. After incubating for 40 min, non-adherent cells were transferred to sterile tubes, washed three times with PBS and counted using an automated cell counter (CELM). These cells were plated and cultured for each experiment.

#### Melanoma cells

Murine melanoma cells (B16F10), permanently maintained in the Department of Cellular Biology, Universidade Federal do Paraná, were used for this study. Cells were maintained in DMEM supplemented with 10% FBS containing 1 U/ml penicillin, 1 μg/ml streptomycin, and 2.5 μg/ml amphotericin at 37°C in a humidified 5%CO_2 _atmosphere.

### Experimental design

The following culture conditions were compared to systematically evaluate the action of the CHM:

B16F10 – melanoma cells only

B16F10/Ly – melanoma cells co-cultured with lymphocytes (Ly)

B16F10/Mϕ – melanoma cells co-cultured with macrophages (Mϕ)

B16F10/Ly-Mϕ – melanoma cells co-cultured with Ly that were previously co-cultured with Mϕ

Mϕ/Ly – Mϕ co-cultured with Ly

Mϕ/Ly* – Mϕ co-cultured with Ly without cell contact (0.4 μm insert)

Each culture condition was divided into two groups: (1) CHM-treated, and (2) untreated (control). A 20% dose was used based on a previously established standard treatment protocol [5,6,9].

### Co-culture assays

All co-culture assays were 48 h in duration, and were repeated at least three times in triplicate. For Mϕ/Ly and Mϕ/Ly* co-culture conditions, mesenteric lymph node lymphocytes (5 × 10^6 ^cells/ml) and macrophages (10^6 ^cells/ml) were incubated for 24 h in the presence (treated) or absence (control) of CHM. For the Mϕ/Ly* culture condition, cells were cultivated in transwell plates (0.4-μm pore size; Costar, Cambridge, MA), with macrophages in the lower chamber and lymphocytes in the upper chamber; both chambers shared the same medium and were thus exposed to the same treatment.

Lymphocytes previously co-cultured with macrophages for 24 h were isolated and incubated with 5 × 10^5 ^cells/ml B16F10 cells (B16F10/Ly-Mϕ culture condition) in 24-well culture plates and then cultured for 48 h, with or without treatment.

### Light microscopy

Cells grown under the culture conditions, B16F10, B16F10/Ly, B16F10/Mϕ and B16F10/Ly-Mϕ, with or without CHM treatment, were evaluated morphologically to optimize culture procedures. The cells were plated on culture plates with cover slips [14,15], and maintained as described above. After 48 h, cells were rinsed with PBS, fixed in Bouin, stained with Giemsa, dehydrated and mounted with Entellan. Adherent cells were observed by light microscopy using a Nikon Eclipse E200 microscope. Melanoma cell density was quantified in B16F10/Ly-Mϕ culture condition by ImageJ software (NIH) selection and pixel area quantification.

### Scanning electron microscopy (SEM)

Culture conditions selected based on optical microscopic screening of B16F10, Mϕ/Ly and B16F10/Ly-Mϕ (treated or untreated), were observed by SEM. After culture, the cells were fixed with 2.5% glutaraldehyde (0.1 M cacodylate buffer, pH 7.2), washed and post-fixed in 1% OsO_4 _for 30 min in the dark at room temperature [14,15]. After washing, the cells were dehydrated using increasing ethanol concentrations. Cells were CO_2 _critical point dehydrated, metallized and observed using a JEOL JSM-6360 LV SEM at the Centro de Microscopia Eletrônica at the UFPR.

### Fluorescence microscopy

The apoptosis rate of melanoma cells was determined in the B16F10/Ly-Mϕ culture condition (with and without CHM treatment) selected based on observations from optical and electron microscopy. DNA cleavage of adherent lymphocytes and B16F10 cells was measured by TUNEL (Terminal deoxynucleotidyltransferase dUTP Nick-End Labeling) assay using the APO-DIRECT TdT-mediated FITC-dUTP nick-end labeling kit as described by the manufacturer (BD Pharmingen). After treatment, adherent cells were fixed with 1% paraformaldehyde in PBS (pH7.4) and then stored in 70% ethanol at -20°C until stained. DNA strand breaks were labeled by incubating cells with 50 μl TUNEL reaction mixture containing TdT and FITC-dUTP in binding buffer for 60 min at 37°C. Cells were then washed and analyzed in an epifluorescence microscope DMLS2 (LEICA). Approximately 100 cells were counted for each cover slip, and a total of about ten cover slips were observed and counted for each treatment and time. Mean percentage data were transformed as described in Statistical analysis.

### Flow cytometry

For immunophenotypic analyses, an aliquot of lymph node cells (10^6^) was incubated with anti-CD3/FITC and anti-CD4/PE, anti-CD8/PE, anti-CD19/PE or Pan-NK/PE (BD Pharmingen) antibodies in PBS/1%FBS for 30 min, and washed three times with PBS. Co-cultured lymphocytes from the culture conditions, Mϕ/Ly, Mϕ/Ly* and B16F10/Ly-Mϕ (treated or untreated), were used to determine CD25 expression and cell viability. These cells (10^6^) were incubated with anti-CD25/FITC (BD Pharmingen) in PBS/1% FBS for 30 min, washed three times with PBS and stained with 7-AAD (BD Pharmingen) for 10 min. For antibody specificity, see Table 1. The culture condition Mϕ/Ly* was used to investigate the influence of macrophages on lymphocytes activation in the absence of cell contact. For each sample, 10^5 ^cells were acquired by CellQuest software (BD) using a FACSCalibur (BD) flow cytometer; the experiments were analyzed with WinMDI 2.9 software.

**Table 1:** Surface markers and viability stain. All antibodies and 7-AAD used were purchase from BD Pharmingen.

Marker	Main marked cells
CD3	T lymphocytes
CD4	Th lymphocytes
CD8	Tc lymphocytes
CD19	B lymphocytes
PanNK (DX5)	NK lymphocytes
CD25	Activated lymphocytes
7-AAD	Non-viable cells

### Statistical analysis

Percentage data obtained from apoptosis assays and flow cytometry analyses were transformed to conform to a normal distribution using the equation, *transformed data *= . Statistical significance of transformed data was determined using a one-way analysis of variance (ANOVA). Statistical significance is presented at the P < 0.05 (*) and P < 0.01 (**) levels. Data are representative of three independent experiments.

## Results

### Altered melanoma cell morphology

**Figure 1:**
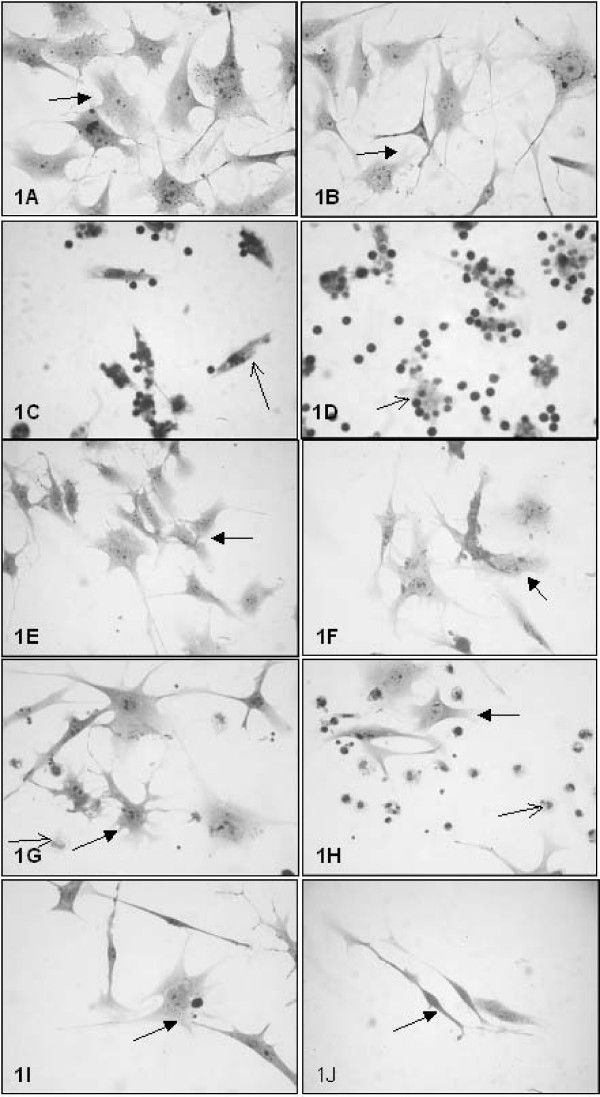
**Microphotographs of control and treated cultures**. The cells were fixed, stained with Giemsa, and observed under a light microscope. Left column: control culture conditions; right column: treated culture conditions. (A and B) B16F10 cells; (C and D) Mϕ/Ly; (E and F) B16F10/Ly; (G and H) B16F10/Mϕ; (I and J) B16F10/Ly-Mϕ. Original magnification: 40× objective for all figures. Black arrow: B16F10 cells; thin black arrow: macrophages.

An examination of cell cultures by light microscopy showed no morphological differences between B16F10 cells treated with the CHM and untreated control cells (Fig 1A and 1B). Treated macrophages and lymphocytes exhibited a greater degree of interaction than did control cells (Fig 1C and 1D).

The melanoma cells from the culture conditions, B16F10/Mϕ and B16F10/Ly, were smaller, indicating a discrete response of the melanoma monolayer to macrophages and lymphocytes (Fig 1E – 1H). This response appeared to be somewhat increased in B16F10/Mϕ and B16F10/Ly cultures treated with the CHM, but the difference was not quantified.

**Figure 2:**
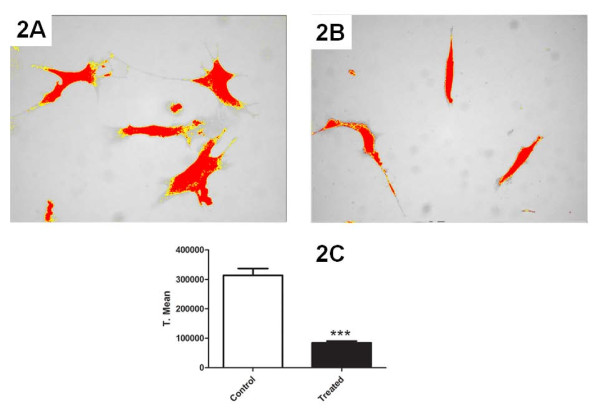
**Micrographs of B16F10 cell density after B16F10/Ly-Mϕ culture condition of control and treated cells respectively (A and B)**. Cell density was evaluated by cell area selection by ImageJ software and posterior pixel area quantification and comparison, showing a decrease of melanoma cell density on this culture condition (C). Original magnification: 40× objective for all figures.

The morphology of cells in the B16F10/Ly-Mϕ culture condition was dramatically altered by the CHM treatment. Lymphocytes previously in contact with macrophages and treated with the CHM were activated in some manner such that they were capable of destroying B16F10 melanoma cells, resulting in a lower density monolayer of melanoma cells (Fig 1I and 1J). Also the quantified melanoma cell density was significant decreased after treatment (Fig 2A–C).

### Induction of cell clustering

**Figure 3:**
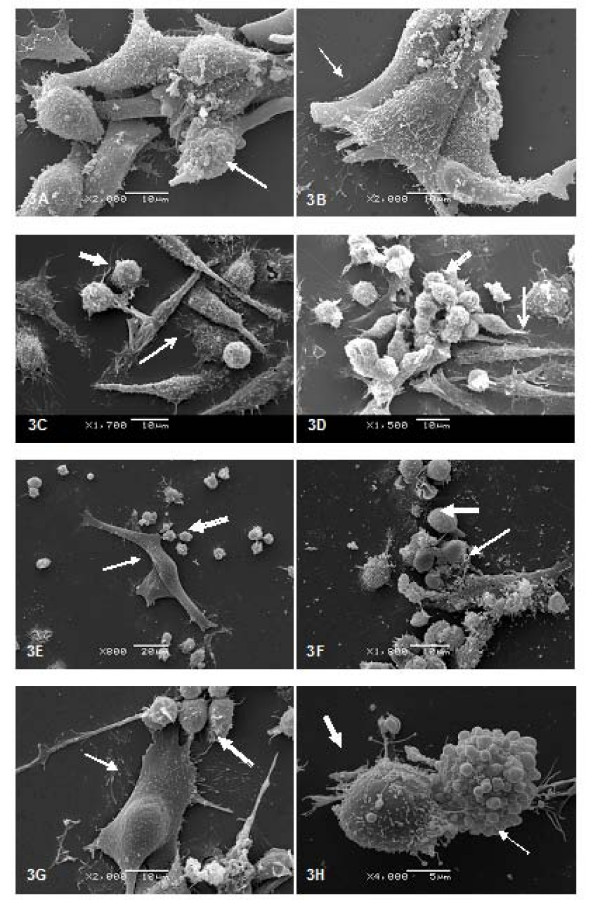
**Scanning electron micrographs of control and treated cultures**. Left column: control culture condition; right column: treated culture condition. (A and B) B16F10 cells after 48 h in culture; (C and D) Mϕ/Ly co-culture; (E and G) untreated B16F10/Ly-Mϕ; (F and H) CHM-treated B16F10/Ly-Mϕ. White arrow: B16F10 cell; thin white arrow; macrophages; Open-headed arrow; lymphocytes.

An examination of cell cultures by SEM showed that B16F10 melanoma cells grew as a monolayer of large and spreading cells with many protrusions. This was true of cells cultured with and without the CHM treatment (Fig 3A–B).

In Mϕ/Ly co-cultures, there was a clear physical interaction between the two cell types (Fig 3C–D). These interactions were enhanced by treatment with the CHM, which resulted in clustering of lymphocytes on macrophages (Fig 3D).

The B16F10/Ly-Mϕ culture condition selected from optical microscopy screening exhibited a decreased density of melanoma cells, which failed to form a monolayer (Fig 3E–H). However, treatment of the same culture condition with the CHM increased the reactivity of lymphocytes towards melanoma cells, resulting in the destruction of virtually all B16F10 cells (Fig 3F). Some cells in the CHM-treated B16F10/Ly-Mϕ culture condition exhibited blebs characteristic of apoptosis (Fig 3H).

Increased lymphocyte viability and expression of the activation marker, CD25, in CHM-treated Mϕ/Ly co-cultures

Flow cytometry was used to identify the proportion of lymphocyte subsets in the mesenteric lymph node cell population before co-culturing with macrophages. As expected, the predominant cell population in murine mesenteric lymph nodes was CD3^+^CD4^+ ^(Table 2).

**Table 2:** Lymphocyte subpopulations in mesenteric lymph nodes. The mean percentage of each cell type determined by immunophenotyping assay is shown.

Markers	%
	
CD3CD4 – Th lymphocytes	44.85
CD3CD8 – Tc lymphocytes	10.65
CD19 – B lymphocytes	9.86
PanNK – NK lymphocytes	3.95
Unstained cells	30.69

**Figure 4:**
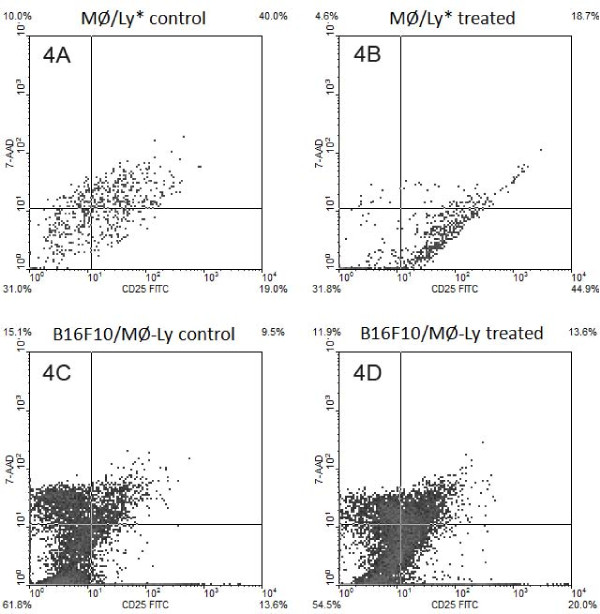
**CD25 expression and viability of lymphocytes after co-culture**. (A and B) Lymphocytes co-cultured with macrophages for 24 h (treated or untreated) without cell contact (0.4 μm insert). In Ly/Mg* was observed a higher amount of unviable (7-AAD^+^) Ly cells in control group (Fig. A). Unviable cells were not included in CD25 analysis for to keep comparison between viable CD25+ cells; (C and D) melanoma cells co-cultured with lymphocytes previously co-cultured with macrophages. Percentage of CD25 and viability (7-AAD^low^) subpopulations in lymphocytic cells are shown at the quadrant extremities.

**Figure 5:**
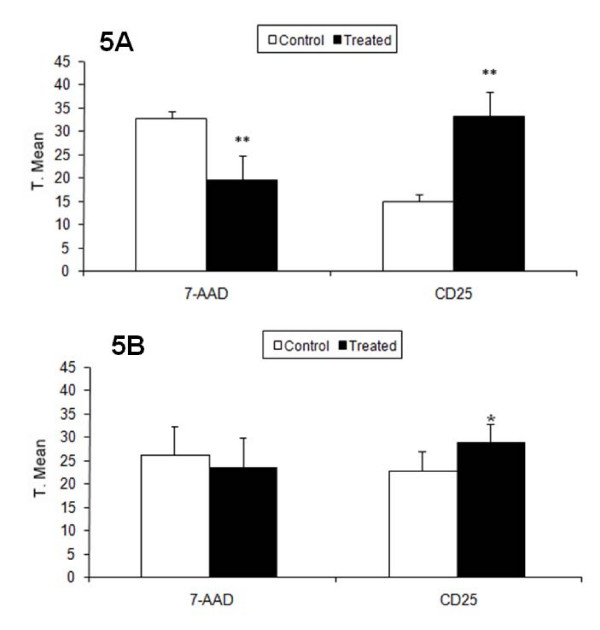
**Lymphocyte CD25 expression and viability (7-AAD^low^) after macrophage co-culture**. (A) Lymphocytes co-cultured with macrophages for 24 h (treated or untreated) without cell contact (0.4 μm insert). CD25 expression and viability significantly increased after treatment. In Ly/Mg* was observed a higher amount of unviable (7-AAD^+^) Ly cells in control group. Unviable cells were not included in CD25 analysis for to keep comparison between viable CD25+ cells. (B) Melanoma cells co-cultured with lymphocytes previously co-cultured with macrophages. Control lymphocyte CD25 expression and viability significantly decreased after 24 h. Y-axis: transformed mean.

The expression of CD25, a marker of activated lymphocytes, was evaluated after co-culturing. In the Mϕ/Ly culture condition, in which both cell types were in direct contact with one another, CD25 expression and viability were similar for lymphocytes with and without the CHM treatment. After removing lymphocytes from co-cultures and placing them with B16F10 cells (culture condition B16F10/Ly-Mϕ), lymphocytes from the Mϕ/Ly* culture condition, when cells was cultured in a Transwell system, and could not contacted with one another, the treatment allowed a significant increase in cell viability and CD25 expression when compared with respective control (Fig 4A and 4B; Table 3; Fig 5A). CD25 expression and viability increased, as did the effectiveness of lymphocytes against melanoma cells (Fig 4C–D; Table 3; Fig 5B).

**Table 3:** CD25 expression in macrophage-stimulated lymphocytes in contact with B16F10 cells.

Mϕ/Ly			
7-AAD viability			

	Mean	Transformed mean	P/F

Control	193	13.927	0.28
	
Treated	179	12.898	

CD25 expression			

	Mean	Transformed mean	P/F

Control	1260.33	35.23	0.72
	
Treated	1150	33.788	

Mϕ/Ly*			

7-AAD viability			

	Mean	Transformed mean	P/F **

Control	1074.66	32.708	0.009
	
Treated	395.67	19.647	

CD25 expression			

	Mean	Transformed mean	P/F **

Control	238.666	14.832	0.007
	
Treated	1119	33.323	

**B16F10/Ly-Mϕ**			

7-AAD viability			

	Mean	Transformed mean	P/F

Control	2368.7	48.3168	0.899
	
Treated	2985.583	54.0464	

CD25 expression			

	Mean	Transformed mean	P/F *

Control	2295.8	59.6266	0.0113
	
Treated	3710.416	47.2793	

Percentage data, obtained from light microscopy analysis, was transformed as described in Materials and Methods. Statistical significance of transformed data was determined by a single-factor ANOVA. Data are representative of three independent co-culture experiments.

### Induction of apoptosis

**Figure 6:**
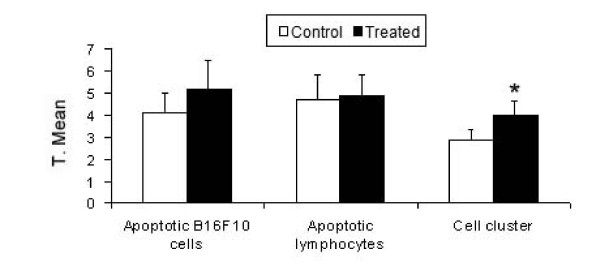
**Apoptotic cells detected by TUNEL assay and analyzed by a one-way ANOVA**. Cell cluster formation was highly significant (P < 0.01), and apoptotic melanoma cell numbers were higher in treated cultures. Y-axis: transformed mean.

Based on morphological evidence of apoptosis by electron microscopy, we selected the B16F10/Ly-Mϕ culture condition for a more thorough analysis by TUNEL assay. Using this technique, lymphocytes were distinguished from melanoma cells by size and nuclear morphology. All cell nuclei were stained red by propidium iodide and only apoptotic cells were stained green by the TdT-mediated FITC-dUTP nick-end labeling. Apoptosis was quantified in both cell types, as well as in the lymphocyte clusters attached to melanoma cells (Table 4), and the functional activity of macrophage-co-cultured lymphocytes against melanoma cells was analyzed by a one-way ANOVA. The degree of cell-cluster formation (Fig 6) was significantly higher with the CHM treatment (**P < 0.01), and the number of apoptotic B16F10 cells was greater.

**Table 4:** Quantification of TUNEL assay of B16F10/Ly-Mϕ culture conditions.

Apoptotic B16F10 cells			
	Mean	Transformed mean	P/F

Control	17	4.0945	0.0708
	
Treated	27.6666	5.1652	

Apoptotic lymphocytes			

	Mean	Transformed mean	P/F

Control	24.75	4.6772	0.6539
	
Treated	22.11	4.9067	

Cell cluster			

	Mean	Transformed mean	P/F **

Control	7.875	2.8636	0.00006
	
Treated	15.3333	3.9635	

Percentage data, obtained from light microscopy analysis, were transformed as described in Materials and Methods. Statistical significance of transformed data was determined by a single-factor ANOVA. Data are representative of three independent experiments.

## Discussion

Tumor cells use multiple mechanisms to escape detection and elimination by the immune system, prompting the development of chemotherapeutic drugs that harness both humoral and cellular immunity to target malignant cells. Innovative immunotherapy approaches include the use of immunotoxins to eliminate regulatory T cells (thereby allowing tumor-specific T cells to be activated), monoclonal antibodies to inhibit immunosuppressive cell signaling, and *exvivo-*expanded tumor-specific T cells in combination with chemotherapy [16]. There is little basic research on the effectiveness of homeopathic treatments in cancer, and the few studies that have been performed are limited in scope. One recent study examined the anti-tumor effect of the homeopathic agent, Lymphomyosot, in a B16F10 *invivo *model [17]. Previous results in mice showed that CHM treatment significantly reduced sarcoma size, infiltration of lymphoid cells, and occurrence of granulation tissue and fibrosis surrounding the tumor, and enhanced immune surveillance and promoted tumor regression [10].

The CHM that we have used here is neither toxic nor mutagenic. Our results show that it induces lymphocyte activation against B16F10 cells, primarily under conditions in which lymphocytes are stimulated by macrophages co-cultured (culture conditions Mϕ/Ly) in the presence of the CHM. These lymphocytes were activated by macrophage stimulation, even in the absence of cell contact. Electron microscopy is a powerful tool for gaining insight into the actions of homeopathic medicaments in cancer [18], providing images that show the reduction of melanoma cell density, membrane disruption, and altered morphology of tumor cells. The cytotoxic effects of macrophage-stimulated lymphocytes against B16F10 in a liquid culture system were also evident in the form of apoptotic cells detected by fluorescence and clearly seen in SEM images (Fig 6 and Fig 3H) and by melanoma cell density quantification (Fig 2). Although there was an apparent increase in apoptotic melanoma cells after Mϕ/Ly interaction, this difference was not significant, possibly reflecting the ability of melanoma cells to suppress apoptosis by over-expressing anti-apoptotic factors [19]. Our results showed that lymphocytes exerted a greater tumoricidal effect not only by inducing apoptosis but also by causing cell lyses. This is consistent with the fact that lymphocytes can kill foreign or tumor cells by other cytotoxic mechanisms, such as release of granzyme B or perforin [20]. The B16F10/Mϕ culture condition did not show increased effectiveness, probably because B16 melanoma cells are capable of inhibiting macrophage activation [21].

Macrophages and dendritic cells both serve important immunomodulatory functions, initiating a primary immune response and also activating lymphocytes. Macrophages can also regulate the intensity of the T-cell response to a pathogen [22,23]. Tumor progression in cancer may not simply result from the absence of an immune response, but rather from the inability of effector immune cells to control or destroy the tumor cells. The interaction between antigen-presenting cells (APCs), like dendritic cells and macrophages, and T cells is characterized by a bidirectional exchange of signals that can result in activation and maturation of effector T lymphocytes [24]. T lymphocytes have spontaneous anti-tumor activity and are capable of killing tumor cells, but the anti-tumor T cell can become inactive over time as a result of tumor-escape mechanisms [25]. Activation of T cells is dependent on APC-interaction mechanisms, and requires the interaction of MHC complexes with TCR, co-stimulatory molecules and/or mediators such as the cytokine, IL-2. Expression of the IL-2 receptor α-chain (CD25) in activated lymphocytes confers greater effector capacity and cellular proliferation [26,27]. Our results confirm this, as evidenced by the fact that treatment with the CHM increased lymphocyte expression of CD25 and lymphocyte viability in conjunction with enhanced effectiveness against melanoma cells and showed to be important to keep the lymphocyte viability in co-culture without contact with macrophages.

Since this medication acts primarily through activation of macrophages [4,9], it is possible that, after being activated, macrophages modulate lymphocyte activation through membrane interactions or secretion of cytokines, such as IL-2, and thereby improve antitumor activity [28,29]. Other signaling pathways might also be involved in this process. Lymphocyte clustering on B16F10 was significantly increased in CHM-treated Mϕ/Ly co-cultures, indicating that macrophage stimulation promoted the interaction of lymphocytes with tumor cells. Many cancers and all melanomas express B7-H1 membrane proteins, which inhibit lymphocyte responses by directly inducing lymphocyte apoptosis. Pre-activated lymphocytes are better prepared to respond to this type of cancer defense [30]. Tumor cells can manipulate the immune microenvironment to their advantage, for example, by promoting TNF-α secretion. TNF-α is rarely cytotoxic to tumor cells and promotes cancer growth, invasion and metastasis, and controls the infiltration of antitumor lymphocytes [31]. This CHM decreased TNF-α production under pathological conditions, and exerted a positive immunomodulatory function by activating macrophages [4,9]. Individual lymphocytes appear not to be activated by the treatment, indicating the importance of immune cell interactions in general and macrophages in particular in CHM-mediated lymphocyte activation.

## Conclusion

In summary, the present study allows us to conclude that this CHM indirectly activates lymphocytes through interaction with macrophages, even without direct cell-cell contact. The co-culture of macrophages and lymphocytes in the presence of the CHM promoted immunostimulation of lymphocytes, resulting in enhanced tumoricidal performance against a very aggressive lineage of melanoma cells. In addition, the lymphocytes activated by the treatment destroyed growing cancer cells more effectively than did control lymphocytes. Finally, these findings suggest that this medication is a promising combination-therapy candidate, which may enhance the efficacy of conventional medicines by improving the immune response or even induce dormancy in tumor cells.

## Competing interests

The authors declare that they have no competing interests.

## Authors' contributions

FSFG designed and performed all experiments and drafted the manuscript. APRA, BC, CCO, LFA and SMO collaborated on cell culture and microscopy experiments. LD and EST acquired and analyzed the microscopy data. JG performed the statistical analyses. DFB supervised all experiments and manuscript writing. All authors have read and approved the final manuscript.

## Pre-publication history

The pre-publication history for this paper can be accessed here:

http://www.biomedcentral.com/1471-2407/9/293/prepub
